# The Impact of High-Temperature Stress on Gut Microbiota and Reproduction in Siberian Hamsters (*Phodopus sungorus*)

**DOI:** 10.3390/microorganisms12071426

**Published:** 2024-07-13

**Authors:** Wenjing Shen, Peng Gao, Kunying Zhou, Jin Li, Tingbei Bo, Deli Xu

**Affiliations:** 1 School of Grassland Science, Beijing Forestry University, Beijing 100083, China; 15257808257@163.com (W.S.); gaopeng2023@bjfu.edu.cn (P.G.); 2 School of Life Sciences, Qufu Normal University, Qufu 273165, China; zky7665@163.com (K.Z.); 17861824275@163.com (J.L.)

**Keywords:** Siberian hamsters, gut microbiota, reproduction, heat stress

## Abstract

Global warming has induced alterations in the grassland ecosystem, such as elevated temperatures and decreased precipitation, which disturb the equilibrium of these ecosystems and impact various physiological processes of grassland rodents, encompassing growth, development, and reproduction. As global warming intensifies, the repercussions of high-temperature stress on small mammals are garnering increased attention. Recently, research has highlighted that the composition and ratio of gut microbiota are not only shaped by environmental factors and the host itself but also reciprocally influence an array of physiological functions and energy metabolism in animals. In this research, we combined 16S rRNA high-throughput sequencing with conventional physiological assessments, to elucidate the consequences of high-temperature stress on the gut microbiota structure and reproductive capacity of Siberian hamsters (*Phodopus sungorus*). The results were as follows: 1. The growth and development of male and female hamsters in the high-temperature group were delayed, with lower body weight and reduced food intake. 2. High temperature inhibits the development of reproductive organs in both female and male hamsters. 3. High temperature changes the composition and proportion of gut microbiota, reducing bacteria that promote reproduction, such as *Pseudobutyricoccus, Ruminiclostridium-E*, *Sporofaciens*, *UMGS1071*, and *CAG_353*. Consequently, our study elucidates the specific impacts of high-temperature stress on the gut microbiota dynamics and reproductive health of Siberian hamsters, thereby furnishing insights for managing rodent populations amidst global climatic shifts. It also offers a valuable framework for understanding seasonal variations in mammalian reproductive strategies, contributing to the broader discourse on conservation and adaptation under changing environmental conditions.

## 1. Introduction

Mammalian reproductive functions are governed by environmental cues such as photoperiod, temperature, and nutrition [1] (Kumar, 1997). Seasonal variations in growth and reproductive efforts represent adaptive mechanisms employed by temperate region animals in response to environmental seasonality, serving as strategies for survival and propagation. These adaptations encompass multifaceted changes in morphology, physiology, and behavior. Temperature, as a key abiotic regulator, plays a pivotal role in orchestrating the seasonal reproductive patterns of animals. Elevated temperatures can exert diverse impacts on animal fecundity, including the attenuation of reproductive capabilities in organisms ranging from fruit flies to cattle [2,3,4].

Amidst climate change, heat stress incidents pose an augmented threat to wild rodents, necessitating frequent encounters with thermal challenges [5,6]. Rodents maintain a relatively constant body temperature, thereby furnishing a stable thermal milieu for the bulk of their biochemical processes [7]. Prolonged heat stress, however, can disrupt this thermal equilibrium and modify strategies for thermoregulation [8,9]. The physiological, behavioral, and reproductive repercussions of heat stress on rodents have garnered substantial research interest [10,11,12]. Conversely, the implications of climate change on gut microbiota dynamics in relation to their hosts remain underexplored [13,14]. Given the potential of microbial symbionts to modulate host behavior and physiology, understanding this interaction may be pivotal in host adaptation to climatic shifts. Existing studies on heat stress-induced changes in gut microbiota primarily concentrate on domestic livestock and poultry [15,16], leaving a significant gap in knowledge regarding its effects on wild animals.

The Siberian hamster (*Phodopus sungorus*) belongs to the Rodentia order, Cricetidae family. It is mainly distributed in the northern part of Hebei Province and most parts of Inner Mongolia Autonomous Region. In the natural environment, the hamster faces large seasonal changes in temperature, and the thermal neutral zone (TNZ) of Siberian hamsters is 23–33 °C; they tolerate severe cold stress but are less able to withstand heat [17]. The Siberian hamster is a typical seasonal breeding animal. There have been many studies on the gut microbiota of Siberian hamsters [18,19,20]; for example, one study has found that the photoperiod regulates the gonadal indicators and gut bacterial composition of male Siberian hamsters [21], and is driven by the pineal gland [20]. A study on Siberian hamsters showed that cold acclimation decreased body weight and gonadal mass [22]. At present, the effects of high environmental temperature on the gut microbiota and reproduction of Siberian hamsters are unknown. Therefore, this study focuses on Siberian hamsters and explores the effects of high-temperature acclimation on their gut microbiota and reproductive performance, as well as their interaction mechanisms. Therefore, both behavioral and microbiological studies on Siberian hamsters are conducive to revealing the molecular mechanisms of their seasonal reproductive activities, greatly promoting population control, rodent pest control, and grassland protection, and providing a strong basis for further research on mammalian seasonal reproduction.

## 2. Materials and Methods

### 2.1. Animals and Experimental Design

All animals were licensed under the Animal Care and Use Committee. Siberian hamsters (*Phodopus sungorus*) (6 weeks old) were housed individually in plastic cages (29 cm × 18 cm × 16 cm), in a constant temperature incubator (MGC-450HP, Yi Heng, Shanghai, China) and were maintained at a room temperature of 23 ± 1 °C, under a photoperiod of 16L:8D. During the test, food and water were provided ad libitum. Water and standard rodent chow (6.2% fat, 35.6% carbohydrate, 20.8% protein, and 17.6 gross energy kJ g^−1^) (Beijing Keao Xieli Feed Co., Beijing, China) were provided. The animal experiments are approved by a regulatory institution and performed according to established guidelines.

To determine the effects of environmental temperature on hamsters, 8 male and 8 female hamsters were stabilized at 23 ± 1 °C for 10 weeks as control groups (Warm male and Warm female), while 8 male and 8 female hamsters were stabilized at 30 ± 1 °C for 10 weeks as treatment groups (High male and High female). After acclimation, collect the feces of hamsters using sterile cryopreservation tubes and dissect them after anesthesia with carbon dioxide. Collect and weigh various organs, including the heart, kidneys, liver, small intestine, BAT, subcutaneous fat, gonadal fat, spleen, cecum, colon, testis, epididymis, ovaries, uterus, and hypothalamus.

### 2.2. Measurements of Body Mass, Food Intake, and Body Temperature

Measure body temperature, body weight, and food weight every 3 days at 9 am. Food intake (g) was calculated by subtracting uneaten food weight from initial food weight and the average daily food intake for 3 days was taken. Body weight and food weight were measured with an electronic balance (Sartorius Model BL 1500 ± 0.1 g). Rectal temperatures were recorded by inserting a temperature probe (TES 1310) 1.5 cm into the rectum daily during the temperature exposure period. 

### 2.3. DNA Extraction and PCR Amplification

Total genome DNA from hamster feces was extracted using the CTAB method. DNA concentration and purity were monitored on 1% agarose gel. According to the concentration, DNA was diluted to 1 ng/μL using sterile water. 16S rRNA genes of distinct regions (16S V3–V4) were amplified used specific primer 341F (5′-CCTAYGGGRBGCASCAG-3′) and 806R (5′-GGACTACNNGGGTATCTAAT-3′) with the barcode. All PCR reactions were carried out with 15 μL of Phusion^®^ High-Fidelity PCR Master Mix (New England Biolabs, Ipswich, MA, USA); 2 μM of forward and reverse primers, and about 10 ng template DNA. Thermal cycling consisted of initial denaturation at 98 °C for 1 min, followed by 30 cycles of denaturation at 98 °C for 10 s, annealing at 50 °C for 30 s, and elongation at 72 °C for 30 s. Finally, 72 °C for 5 min, then mix the same volume of 1XTAE buffer with PCR products and operate electrophoresis on 2% agarose gel for detection. PCR products were mixed in equidensity ratios. Then, the mixed PCR products were purified with Universal DNA (TianGen, Beijing, China).

### 2.4. Libraries Generated and Illumina NovaSeq Sequencing

Sequencing libraries were generated using NEB Next^®^ Ultra DNA Library Prep Kit (New England Biolabs, Ipswich, MA, USA) following manufacturer’s recommendations and index codes were added. The library quality was assessed on the Agilent 5400 (Agilent Technologies Co., Ltd., Beijing, China). At last, the library was sequenced on an Illumina NovaSeq platform and 250 bp paired-end reads were generated.

### 2.5. Bioinformatics Analysis

The analysis was conducted by following the “Atacama soil microbiome tutorial” of Qiime2docs along with customized program scripts (https://docs.qiime2.org/2019.1/ (accessed on 12 January 2024). Briefly, raw data FASTQ files were imported into the format that could be operated by QIIME2 system using QIIME2 (2020. 11) tools import program. Demultiplexed sequences from each sample were quality filtered and trimmed, de-noised, and merged, and then the chimeric sequences were identified and removed using the QIIME2 dada2 plugin to obtain the feature table of amplicon sequence variant (ASV) [23]. Any contaminating mitochondrial and chloroplast sequences were filtered using the QIIME2 feature-table plugin (2023.5). Appropriate methods including ANCOM, ANOVA, Kruskal–Wallis, LEfSe, and DEseq2 were employed to identify the bacteria with different abundances among samples and groups. Diversity metrics were calculated using the core-diversity plugin within QIIME2. Feature level alpha diversity indices, such as observed OTUs, Chao1 richness estimator, Shannon diversity index, and Faith’s phylogenetics diversity index were calculated to estimate the microbial diversity within an individual sample. Beta diversity distance measurements, including Bray–Curtis were performed to investigate the structural variation in microbial communities across samples and then visualized via principal coordinate analysis (PCoA). Co-occurrence analysis was performed by calculating Spearman’s rank correlations between predominant taxa and the network plot was used to display the associations among taxa. In addition, the potential KEGG Ortholog (KO) functional profiles of microbial communities were predicted with PICRUSt [24]. Unless specified above, parameters used in the analysis were set as default.

### 2.6. Measurement of mRNA by Real-Time Quantitative PCR (RT-qPCR)

The total RNA was extracted from the tissue (testis, epididymis, uterus, and hypothalamus) using TRIzol™ LS Reagent (10296028, Thermo Scientific, Waltham, MA, USA), and then, reversed transcription was used to generate cDNA according to supplier specifications (Code No. RR820Q/A/B, TAKARA, Dalian, China). RT-qPCR analysis was carried out as follows: the cDNA samples (2 µL) were used as a template for the subsequent PCR reaction using gene-specific primers (Supplementary Table S1). RTqPCR was performed using Piko Real Software 2.2 (Piko Real 96, Thermo Scientific). Genes related to reproduction and development were measured, including kisspeptin1 (an important regulator involved in the molecular mechanism of mammalian reproduction), TSHβ (Thyroid-stimulating hormone β, regulating male reproduction), TSHR (thyrotropin receptor, regulating male reproduction), and ESRβ (Estrogen Receptor β, regulating female reproduction).

### 2.7. Measurements of Estradiol

Blood of hamsters was stored in a 4 °C freezer overnight, then centrifuged at 4000 RPM for 30 min; the supernatant (serum) was collected and stored at −80 °C. Serum estradiol concentrations were quantified using a 17 beta Estradiol ELISA kit (ab 108667, Abcam, Cambridge, UK) according to the instructions. The minimum detected concentration of the kit was 8.68 pg/mL.

### 2.8. Statistical Analysis

Statistical analysis was conducted using the SPSS 22.0 software package and GraphPad Prism 9. Differences in body mass, body temperature, and food intake were compared between treatment groups using a repeated-measure ANOVA. Measurements of other indexes were compared using two-way ANOVAs and Kruskal–Wallis test with *p* < 0.05 (* *p* < 0.05, ** *p* < 0.01, *** *p* < 0.001). Results were presented as means ± SEM.

## 3. Results

**Figure 1:**
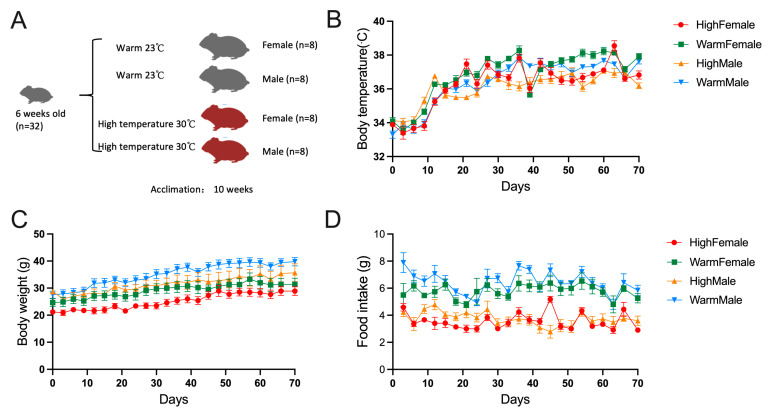
High temperature affects the development of *Phodopus roborovskii:* (**A**) Design of experiment 1. (**B**) Body weight. (**C**) Body temperature. (**D**) Food intake (*n* = 8).

Repeated measures analysis of variance showed that the body temperature of hamsters significantly increased over time (female: *p* < 0.001; male: *p* < 0.001), and there were also significant differences between warm- and high-temperature groups of females (*p* = 0.007), but no significant differences in males (*p* = 0.064; Figure 1B). The weight of hamsters significantly increased over time (female: *p* < 0.001; male: *p* < 0.001), and there were no significant differences between warm- and high-temperature groups (female: *p* = 0.07; male: *p* = 0.167; Figure 1C). The food intake of hamsters significantly changed over time (female: *p* < 0.001; male: *p* < 0.001), and there were also significant differences between warm- and high-temperature groups of female and male, and the food intake of the high-temperature group decreased significantly (*p* < 0.01; Figure 1D). 

**Figure 2:**
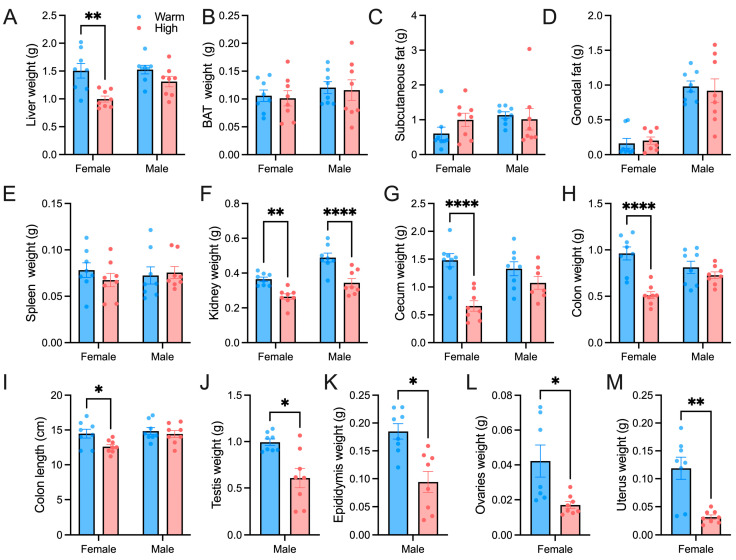
High temperature affects the organ indicators of *Phodopus roborovskii:* (**A**) Liver weight. (**B**) BAT weight. (**C**) Subcutaneous fat weight. (**D**) Gonadal fat weight. (**E**) Spleen weight. (**F**) Kidney weight. (**G**) Cecum weight. (**H**) Colon weight. (**I**) Colon length. (**J**) Testis weight. (**K**) Epididymis weight. (**L**) Ovaries weight. (**M**) Uterus weight. * *p* < 0.05, ** *p* < 0.01, **** *p* < 0.0001. (*n* = 8).

**Figure 3:**
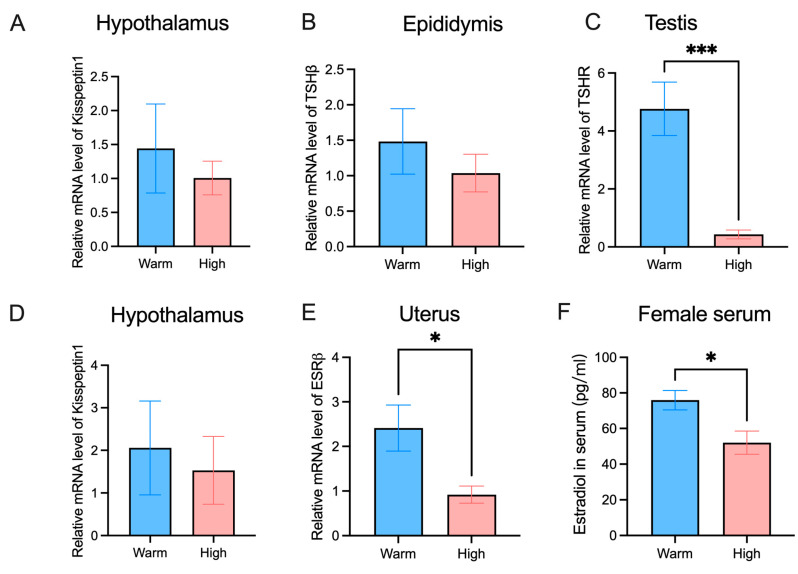
High temperature affects the reproductive level of *Phodopus roborovskii:* (**A**) Expression of Kisspeptin1 in male hypothalamus. (**B**) Relative mRNA level of TSHβ in epididymis. (**C**) Relative mRNA level of TSHR in testis. (**D**) Expression of Kisspeptin1 in female hypothalamus. (**E**) Relative mRNA level of ESRβ in the uterus. (**F**) Concentration of Estradiol in female serum. * *p* < 0.05, *** *p* < 0.001. (*n* = 8).

Some basic anatomical indicators were tested, high-temperature acclimation reduced the liver weight of female hamsters (Figure 2A), but there was no difference in the weight of BAT and white fat (Figure 2B–D). High-temperature acclimation reduced the kidney weight of hamsters (Figure 2F), but there was no difference in spleen weight (Figure 2E). High temperature reduced the weight of the cecum and colon as well as the length of the colon in female hamsters (Figure 2G–I). In male hamsters, we found that high temperature reduced the weight of the testes and epididymis (Figure 2J,K). High temperatures in females reduced ovaries and uterus weight (Figure 2L,M). High temperature decreased the expression of kisspeptin1 in the hypothalamus of males and females (Figure 3A,D). The relative mRNA level of TSHβ in the epididymis was lower in the high-temperature group (Figure 3B). The relative mRNA level of TSHR in the testis was lower in the high-temperature group (Figure 3C). The relative mRNA level of ESRβ in the uterus was significantly lower in the high-temperature group (Figure 3E). We measured the serum estradiol of females. The content of estradiol in the high-temperature group was significantly lower than that in the warm group (Figure 3E).

**Figure 4:**
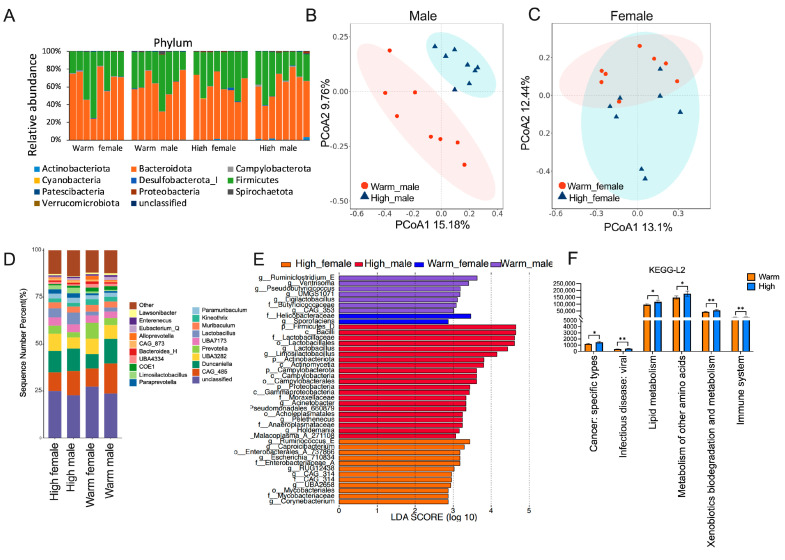
High-temperature-shaped diversity and composition of gut microbiota in male and female hamsters (*n* = 8): (**A**) Taxonomy composition at the phylum level of 4 groups. (**B**) PCoA based on Bray–Curtis distance in male hamsters. (**C**) PCoA based on Bray–Curtis distance in female hamsters. (**D**) Taxonomy composition at the genus level of 4 groups. (**E**) LEfSe identified the most differentially abundant taxa at the different levels among 4 groups (LDA > 2, *p* < 0.05). (**F**) KEGG pathway analysis showing pathways that were significantly different between warm- and high-temperature groups (corrected *p* < 0.05). Data are means ± SEM. * *p* < 0.05, ** *p* < 0.01. (*n* = 8).

To investigate whether high temperature causes changes in the gut microbiota, we collected feces at the end of the experiment. Firmicutes and Bacteroidetes were the most abundant phylum in all samples (Figure 4A). In the warm-temperature group of males, Firmicutes accounted for 38%, while the high-temperature group accounted for 34.5%. For alpha diversity, there was no difference among the four groups (Figure S1A,B, Table S2). For the beta diversity, PCoA analysis based on Bray–Curtis distance, and the “PERMANOVA” test showed significant differences among the four groups (test statistic = 1.304247, *p* = 0.003, Table S3). When only analyzing the environmental temperature effect, the NMDS figure showed a significant difference (Figure S1C). In male hamsters, the two temperatures were clearly separated (Figure 4B). The effect of high temperature on microbial beta diversity in female hamsters is not significant (Figure 4C). The Venn diagram represents the degree of microbial sharing among the four groups (Figure S1D). At the genus level, we also used a summary bar chart to show the difference between the four groups (Figure 4D). Linear discriminant analysis effect size (LEfSe) displayed that the content of *Ruminococcus*, *Caproicibacterium*, *Escherichia*, *RUG12438*, and *Corynebacterium* significantly increased in the high-female group. The content of *Lactobacillus*, *Limosilactobacillus*, *Acinetobacter*, *Pelethenecus*, and Holdemania significantly increased in the high-male group (Figure 4E). Also, *Ruminiclostridium*, *UMGS1071*, *Ventrisoma*, *Ligilactobacillus*, and *Sporofaciens* were lower in high-temperature groups. We found the gut microbiota of the high-temperature group was significantly increased in several functional categories, compared to the warm group. These different functional categories were related to lipid metabolism, other amino acid metabolism, and the immune system (Figure 4F).

**Figure 5:**
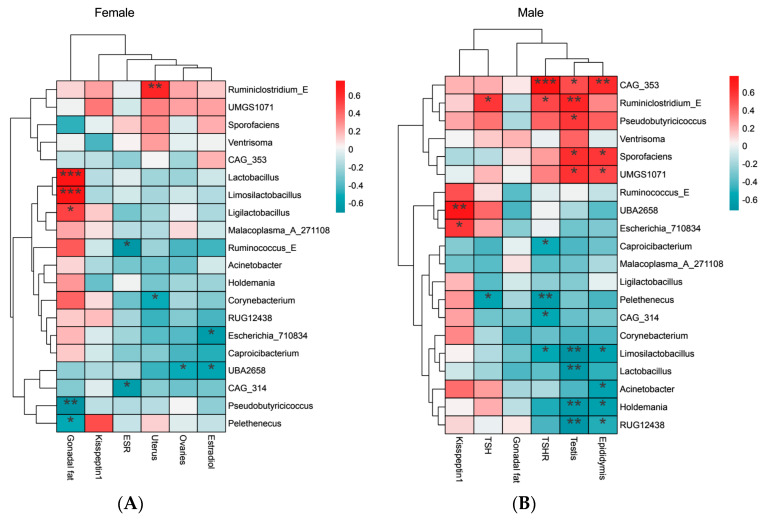
Correlation analysis between different genus abundance and physiological indexes (Spearman): (**A**) Correlation analysis between different genus abundance and reproductive indexes in females. (**B**) Correlation analysis between different genus abundance and reproductive indexes in males. * *p* < 0.05, ** *p* < 0.01, *** *p* < 0.001.

Correlation analysis between different genus abundance and physiological indexes showed Corynebacterium and UBA2658A were negatively correlated with uterus and ovaries weights, *Ruminiclostridium_E* and *CAG_314* were negatively correlated with ESRβ, while Lactobacillus and Limosilactobacillus were positively correlated with gonadal fat weight (Figure 5A). In males, *Lactobacillus*, *Limosilactobacillus*, *Holdemania,* and *RUG12438* were negatively correlated with testis and epididymis weights. These bacteria were more abundant in high-temperature groups (Figure 4E). However, some bacteria lacking in the high-temperature group, such as *Pseudobutyricicoccus* and *Ruminiclostridium_E*, *Sporofaciens*, *UMGS1071*, and *CAG_353* were positively correlated with the weight of the testes and epididymis, and *Ruminiclostridium_E* was positively correlated with the expression of TSHβ (Figure 5B). 

## 4. Discussion

### 4.1. The Inhibitory Effect of High Temperature on the Reproduction of Siberian Hamsters

We investigated the effects of high temperatures on the gut microbiota and reproductive performance of Siberian hamsters. Acclimation to high temperatures led to reduced body weights and food intake in these hamsters, consistent findings observed in both males and females, akin to studies conducted on the Mongolian gerbils (*Meriones unguiculatus*). In our research, at an ambient temperature of 35 °C, the body temperature of Siberian hamsters was slightly lower, aligning with observations in Brandt’s voles and Mongolian gerbils [25]. These species, all small mammals inhabiting desert regions, possess a degree of tolerance to high temperatures. The research suggests that when faced with elevated environmental temperatures, Siberian hamsters can tolerate heat stress by enhancing heat dissipation and lowering their body temperature. This thermoregulatory response may also involve animals licking and spreading saliva over their bodies to augment evaporative cooling, thereby contributing to a decrease in body temperature. Such adaptations highlight their resilience in coping with the harsh conditions prevalent in their arid habitats.

High temperatures have an inhibitory effect on the reproduction of small mammals, with testicular hyperthermia leading to reduced sperm vitality and count, as well as accelerated germ cell apoptosis [26]. Our study revealed that the weights of the testes and epididymides in the high-temperature group of Siberian hamsters were significantly reduced, impairing their reproductive capability. Most mammals have their testes located outside the body cavity to facilitate appropriate thermoregulation. Elevated temperatures negatively impact spermatogenesis in mammals, ultimately resulting in subfertility or infertility [11]. Furthermore, in molecular experiments, we observed decreased expression of hypothalamic kisspeptin1 in male hamsters from the high-temperature group, a gene crucial for sexual development. Mammalian seasonal breeding is regulated by a complex feedback mechanism involving the hypothalamic-pituitary-gonadal (HPG) axis. A pivotal branch of the hypothalamic-pituitary axis is the hypothalamic-pituitary-thyroid (HPT) axis, with the thyroid gland being a vital endocrine organ participating in reproductive activities. Thyroid-stimulating hormone (TSH) is essential for thyroid structure and metabolism, playing a critical role in regulating the normal secretion of thyroid hormones, which, in turn, modulates fundamental life processes such as growth, development, and reproduction. Our findings of lowered TSHβ expression in the epididymis and reduced TSH receptor (TSHR) expression in the testes suggest suppressed reproductive signaling. Abundant evidence indicates that heat stress damages oocytes and the follicles enveloping them. Exposure to high temperatures ten days before estrus is associated with reduced fertility [27]. Studies in rats have demonstrated that heat stress decreases the levels of gonadotropin receptors in granulosa cells and estradiol concentrations [28]. We observed that high temperatures induced reductions in the mass of ovaries and uterus in female Siberian hamsters, alongside lowered kisspeptin1 expression in the hypothalamus, decreased ESRβ expression in the uterus, and significantly reduced serum estradiol levels compared to the control group. Consequently, heat stress suppresses the reproductive capacity of Siberian hamsters, affecting both females and males.

### 4.2. High Temperature Affects the Composition and Function of Gut Microbiota

Microorganisms and other organisms grow within specific temperature ranges and respond to temperatures that deviate from their optimal conditions. The pattern of reduced diversity of host microbiota under experimental warming was also found among mammals, amphibians, and birds [29,30]. This likely reflects the negative effects of extreme temperatures on the physiological functions of the host [31]. Our study intriguingly observed a slight, albeit statistically nonsignificant, increase in the gut microbiota diversity of hamsters post heat stress. This phenomenon might be attributed to the Siberian hamster’s inherent adaptability to its desert habitat, endowing it with a heightened resilience to fluctuations in environmental temperatures. Although homeotherms, or warm-blooded animals, typically maintain a constant body temperature, they are not impervious to the repercussions of environmental heat stress, as evidenced by reduced feed intake, stunted growth, enhanced intestinal permeability, and a heightened risk of systemic infections in livestock reared under hot and congested conditions [32,33]. Many studies have documented alterations in the microbiota profiles of heat-stressed livestock, marked by declines in α-diversity and Firmicutes abundance, accompanied by a surge in Proteobacteria [34,35,36,37,38]. Heat stress is similarly implicated in elevating the risk of bacterial translocation and sepsis in humans, as well as fostering an environment conducive to Gram-negative bacterial infections [39,40]. In our research, a conspicuous effect was noted in male Siberian hamsters, where high temperatures led to a decline in the proportion of Firmicutes. This observation aligns with the concept that body temperature directly or indirectly, through its influence on host feeding behavior or metabolic adjustments, exerts a consistent impact on the gut’s Firmicute population [31]. Notably, our study also unveiled distinct sex-specific responses to heat stress. The gut microbiota of female hamsters displayed increases in *Ruminococcus*, *Caproicibacterium*, *Escherichia*, *RUG12438*, and *Corynebacterium*, while in males, substantial rises were seen in *Lactobacillus*, *Limosilactobacillus*, *Acinetobacter*, *Pelethenecus*, and *Holdemania*. These differences indicate possible interactions between host reproduction and gut microbiome under environmental heat stress.

In addition, diet is a key factor affecting the structure of the gut microbiota. In our experiment, high temperature reduced the food intake of hamsters, which may affect the structure of their gut microbiota. However, according to research on Brandt’s vole, the gut microbiota is mainly affected by temperature rather than food intake [41], and slight changes in the core temperature of the vole can cause changes in the gut microbiota [42]. Therefore, we speculated that the effect of high temperature on the gut microbiota of Siberian hamsters was greater than the effect of reducing food intake.

### 4.3. Regulation of Gut Microbiota on the Reproduction of Siberian Hamsters

Studies have found that the gut microbiota is related to reproduction. For example, male mice consuming *Lactobacilli* exhibit an increase in testicular volume and serum testosterone levels [43]. Feeding elderly male mice with *Lactobacillus reuteri* can restore testosterone to younger levels, providing evidence that the gut microbiota regulates testosterone production and testicular aging [44]. Transplanting the gut microbiota of high-fat diet mice into the intestines of normal mice significantly increases the abundance of Bacteroidetes and Prevotella, while Prevotella is significantly negatively correlated with sperm motility. Transplantation of the microbiota also leads to an increase in pro-inflammatory cytokines in the epididymis of mice [45]. Gut microbiota and their metabolites also play important roles in the female reproductive endocrine system by interacting with estrogen, androgens, insulin, and other hormones [46,47,48,49,50]. In our study, we found that high-temperature acclimation can cause an increase in the content of many bacteria, which are negatively correlated with the weight of reproductive organs. Specifically, *Corynebacterium* and UBA2658A were negatively correlated with uterus and ovaries weights, and *Lactobacillus*, *Limosilactobacillus*, *Holdemania*, and *RUG12438* were negatively correlated with testis and epididymis weights. The bacteria with reduced abundance in the high-temperature group are mostly positively correlated with reproduction, for example, *Pseudobutyricicoccus* and *Ruminiclostridium_E*, *Sporofaciens*, *UMGS1071*, and *CAG_353*. Gut microbiota regulates sex hormone levels by producing enzymes and releases active estrogen by releasing β-glucuronidase to dissociate the estrogen–bile acid complex [51,52]. Our results suggest that the gut microbiota is closely associated with the reproductive genes and hormones in the central and peripheral tissues of hamsters. *Ruminiclostridium_E* was positively correlated with the expression of TSHβ in the epididymis, which was not enriched in high-temperature group hamsters. There are still many imperfections in this study, and in the future, we will continue to conduct long-term temperature acclimation to explore the effects of different temperature gradients on Siberian hamsters. In addition, microbiota transplantation experiments will also be conducted to further explore the impact of gut microbiota on the reproduction of Siberian hamsters.

## 5. Conclusions

Our study found that high-temperature stress leads to a decrease in the reproductive performance of Siberian hamsters, and the diversity and structure of gut microbiota changed over heat stress. Our research shows that Siberian hamsters maintain energy balance under heat stress by reducing host reproductive development, and speculates possible interactions between host reproduction and gut microbiota. In the future, verification experiments should be used to further confirm this correlation.

## Data Availability

The raw sequencing data generated in this study have been deposited in the NCBI Sequence Read Archive under the accession numbers PRJNA1121091.

## Supplementary Materials

The following supporting information can be downloaded at: https://www.mdpi.com/article/10.3390/microorganisms12071426/s1, Figure S1. High temperature shaped diversity and composition of gut microbiota in male and female hamsters (n = 8). Table S1. Gene-specific primers; Table S2. Kruskal-Wallis (pairwise) of alpha diversity; Table S3. Pairwise permanova results of 4 groups.
