# Supplementary material for: The Impact of High-Temperature Stress on Gut Microbiota and Reproduction in Siberian Hamsters (Phodopus sungorus)
Source: Microorganisms. 2024 Jul 13;12(7):1426. doi: 10.3390/microorganisms12071426 (PMC11278997; doi:10.3390/microorganisms12071426)
Supplement: Supplementary file 1 [file microorganisms-12-01426-s001.zip › microorganisms-3084230-supplementary.pdf]

Supplementary

The Impact of High-Temperature Stress on Gut Microbiota and Reproduction in Siberian Hamsters (*Phodopus sungorus*)

Wenjing Shen [1,†], Peng Gao [1,†], Kunying Zhou [2], Jin Li [2], Tingbei Bo [1,*] and Deli Xu [2,*]

[1] School of Grassland Science, Beijing Forestry University, Beijing 100083, China; 15257808257@163.com (W.S.); gaopeng2023@bjfu.edu.cn (P.G.)
[2] School of Life Sciences, Qufu Normal University, Qufu 273165, China; zky7665@163.com (K.Z.); 17861824275@163.com (J.L.)
* Correspondence: botingbei@bjfu.edu.cn (T.B.); xudl1975@163.com (D.X.)
† These authors contributed equally to this work.

Table S1. Gene-specific primers

Gene	Primers
kisspeptin-1（F）	ACTCATCAATGCCTGGGAAAAG
kisspeptin-1（R）	CCGAAGGAGTTCCAGTTGTAGG
TSHβ（F）	GTGCCTATTGCCTAACCATCA
TSHβ（R）	AGGGTAGGAGAAATAAGGAGCA
ESRβ（F）	GCTCAGCCTGTTGGACC
ESRβ（R）	CGCACTTTCCCTCATCC
Actin（F）	CAGGGCGTGATGGTGGGCATGGG
Actin（R）	CCTCAGTGAGCAGCACAGGGT

Table S2. Kruskal-Wallis (pairwise) of alpha diversity

Group 1	Group 2	H	p-value	q-value
High_female (n=8)	High_male (n=8)	0.705882	0.400814	0.792918
Warm_female (n=8)		1.334559	0.247996	0.743987
Warm_male (n=8)		2.161765	0.141482	0.743987
High_male (n=8)	Warm_female (n=8)	0.011029	0.916359	0.916359
Warm_male (n=8)		0.397059	0.528612	0.792918
Warm_female (n=8)	Warm_male (n=8)	0.099265	0.752714	0.903257

Table S3. Pairwise permanova results of 4 groups

Group 1	Group 2	Sample size	Permutations	pseudo-F	p-value	q-value
High_female	High_male	16	999	1.081171615	0.268	0.3216
High_female	Warm_female	16	999	1.286321451	0.055	0.0825
High_female	Warm_male	16	999	1.263556857	0.04	0.08
High_male	Warm_female	16	999	1.52431683	0.006	0.018
High_male	Warm_male	16	999	1.648623656	0.003	0.018
Warm_female	Warm_male	16	999	1.054379622	0.331	0.331

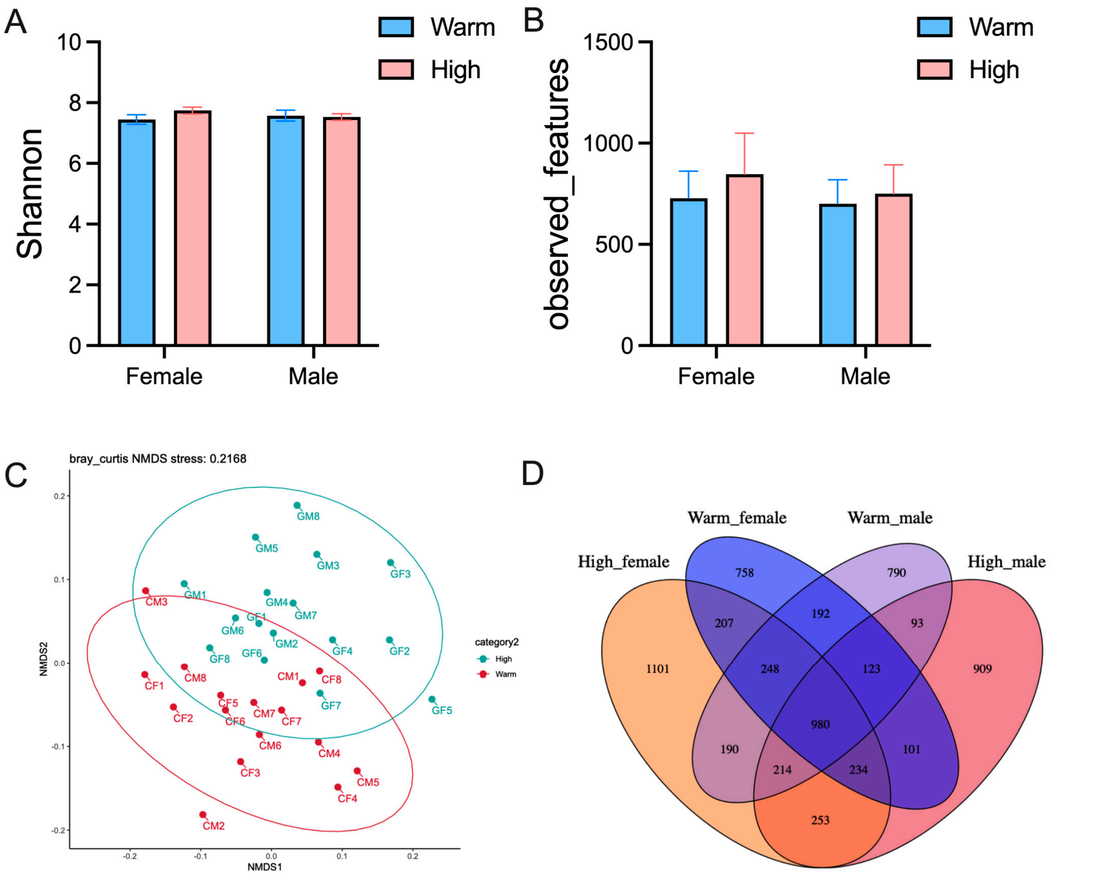

Figure S1. High temperature shaped diversity and composition of gut microbiota in male and female hamsters (n = 8). (A) Shannon diversity of gut microbiota. (B) Observed_features of gut microbiota. (C) NMDS plot of bray_curtis distance of gut microbiota in two groups. (D) Venn diagram of 4 groups. Data are means ± SEM. (n =8).
